# Molecular evidence of *Rickettsia felis *infection in dogs from northern territory, Australia

**DOI:** 10.1186/1756-3305-4-198

**Published:** 2011-10-11

**Authors:** Sze-Fui Hii, Steven R Kopp, Mary F Thompson, Caroline A O'Leary, Robert L Rees, Rebecca J Traub

**Affiliations:** 1 School of Veterinary Science, The University of Queensland, Gatton, Queensland, 4343 Australia; 2 Bayer Animal Health Tingalpa, Brisbane, Queensland, 4343 Australia

## Abstract

The prevalence of spotted fever group rickettsial infection in dogs from a remote indigenous community in the Northern Territory (NT) was determined using molecular tools. Blood samples collected from 130 dogs in the community of Maningrida were subjected to a spotted fever group (SFG)-specific PCR targeting the *omp*B gene followed by a *Rickettsia felis*-specific PCR targeting the *glt*A gene of *R. felis*. *Rickettsia felis omp*B and *glt*A genes were amplified from the blood of 3 dogs. This study is the first report of *R. felis *infection in indigenous community dogs in NT.

## Introduction

Rickettsioses are important emerging vector-borne diseases in humans [1], and some have been reported to infect dogs [2,3]. Rickettsioses that are purported to be endemic in Australia include murine typhus (*Rickettsia typhi*), Queensland tick typhus (*Rickettsia australis*), Flinders Island spotted fever (*Rickettsia honei*), scrub typhus (*Orientia tsutsugamushi*) and Q fever (*Coxiella burnetti*). Cat flea typhus or flea-borne spotted fever, caused by *Ricketsia felis*, which was first described in humans in the USA [4], is an emerging zoonosis that has been reported from throughout the world and was recently reported in a cluster of family members in Victoria, Australia [5].

The cat flea, *Ctenocephalides felis*, is a reservoir and biological vector of *R. felis *[6]. In Australia, *R. felis *DNA was first identified in fleas in 2006 [7]. Very recently, a molecular study in Australia detected *R. felis *in 9% of 100 tested Southeast Queensland (SE QLD) pound dogs, suggesting that dogs may act as mammalian reservoir hosts for *R. felis *[8]. Given the close bond that exists between humans and canines, it is possible that dogs may be a potential source of *R. felis *infection for humans. Dogs are also important in indigenous community life, and it is therefore prudent to better understand the public health risks that may be associated with the human-animal bond in these communities. To this end, we sought to investigate the prevalence of spotted-fever group organisms in dogs from the indigenous community of Maningrida in the Northern Territory (NT), using PCR assays.

## Materials and methods

Blood samples were collected from 130 dogs undergoing sterilisation facilitated by the Animal Management in Rural and Remote Indigenous Communities (AMRRIC) organisation in the indigenous community of Maningrida, NT. Sixty of these dogs were tested in September 2009 and 70 in September 2010. The sampled cohort of dogs was represented by 56 entire males and 69 entire females. Data on gender were unavailable for 5 dogs. One mL of whole blood was obtained from each dog by venipuncture, and the blood was immediately transferred to a QIAcard FTA^® ^Four Spots (Qiagen, Hilden, Germany) to facilitate preservation of DNA during transport.

DNA extraction from blood applied to FTA cards was carried out using the MasterPure™ DNA purification kit (Epicentre Biotechnologies, Madison, USA), following the protocol described by Abd Rani et al. [9].

All extracted DNA was subjected to a previously described PCR assay which amplifies a portion of the *omp*B gene in SFG rickettsiae [10]. DNA extracted from a *Rickettsia conorii *culture obtained from the Australian Rickettsial Reference Laboratory was used as a positive control, whilst nuclease- free water (NFW) was used as a negative control. Blood samples found to be positive for SFG rickettsial DNA were further subjected to a species-specific nested PCR for *R. felis*, which amplified the *glt*A gene (Table 1). *Rickettsia felis *specific primers were designed by aligning *glt*A sequences retrieved from GenBank of closely related rickettsial species (*R. felis*, [GenBank: CP000053]; *R. australis*, [GenBank: U59718]; *Rickettsia_rickettsii*, [GenBank: U59729]; *R. typhi*, [GenBank: U59714]) using CLUSTALW program http://www.genome.jp/tools/clustalw/. Nested PCR primers were manually designed by selecting a region specific to *R. felis *(Table 1). However, the potential cross-reaction of this newly designed PCR was not tested with other *Rickettsia spp*. This PCR that was developed relies on confirming diagnosis by DNA sequencing to ensure there is no cross reactivity with closely related organisms.

**Table 1:** Sequences and primer sets for the amplification of a partial region of the *omp*B gene of SFG rickettsiae and *glt*A gene of *R. felis*

Gene	Primer name and sequence (5'-3')	Product size
*omp*B	*omp*B-F (CGACGTTAACGGTTTCTCATTCT) *omp*B-R (ACCGGTTTCTTTGTAGTTTTCGTC)	252 bp [10]

*glt*A	gltA-F1 (GCAAGTATTGGTGAGGATGTAATC) gltA-R1 (CTGCGGCACGTGGGTCATAG) gltA-F2 (GCGACATCGAGGATATGACAT) gltA-R2 (GGAATATTCTCAGAACTACCG)	654 bp

Primary PCR amplification of the *glt*A gene of *R. felis *was performed in a 25 μl reaction mixture containing 2 μl of DNA, 5 μl 5× PCR buffer, 200 μmol/L dNTP, 2.0 mmol/L MgCl_2_, 0.5 units of GoTaq polymerase (Promega, Madison, WI, USA), 10 pmol of each forward and reverse primer made to the final volume with NFW. Two μl of distilled water and *R. felis *DNA isolated from an infected cat flea were used as negative and positive control, respectively. PCR cycling conditions comprised an initial activation step at 95°C for 2 min, followed by 40 cycles of 95°C for 45 s, 63°C for 30 s and 72°C for 45 s with a final extension step of 72°C for 7 min. The secondary round of PCR amplification was performed as for the primary round except that the annealing temperature was 59°C.

All amplified PCR products of both SFG rickettsial and *R. felis*-specific PCR were visualised via transillumination on 1.6% agarose gel stained with SYBR^® ^Safe (Invitrogen, Eugene, USA) DNA gel stain. All PCR products of the correct size were purified using QIAquick™ PCR Purification Kit (Qiagen, Hilden, Germany) according to manufacturer's protocol and subjected to DNA sequencing using both forward and reverse primers. DNA sequences were assessed for homology with those in GenBank using the NCBI Blast program http://blast.ncbi.nlm.nih.gov/Blast.cgi.

This project was approved by the University of Queensland Animal Ethics Committee.

## Results

Of the total 130 tested samples, three (2.3%) were SFG rickettsial PCR-positive and DNA sequences were > 99% homologous with *R. felis omp*B gene [GenBank: GQ385243]. Subsequent *R. felis*-specific nested PCR determined all three SFG positive dogs to harbour *R. felis *DNA. Sequences were 100% homologous to the *R. felis glt*A gene [GenBank: CP000053].

## Discussion

This is the first study to report the presence of *R. felis *infection in indigenous community dogs in the NT. In Australia, *R. felis *was first identified in fleas collected from dogs and cats in Western Australia in 2006 [7]. Recently, 19.8% of fleas collected from cats in Brisbane, Sydney and Melbourne [11] and 9% of pound dogs in SE QLD in Australia were reported to harbour *R. felis *DNA as identified by PCR [8]. Cat flea typhus was also reported in a cluster of human patients in Victoria [5]. The findings of these studies supported the presence of a peridomestic cycle and wide geographical distribution for this pathogen in Australia.

In the previous molecular study, the infection of *R. felis *in pound dogs in SE QLD was confirmed by PCR assay targeting only the *omp*B gene and confirmed by phylogenetic analysis. In this study, the *glt*A gene of *R. felis *in dogs' blood was amplified in addition to the *omp*B gene by a novel *R. felis*-specific nested PCR. This study strengthens the evidence for dogs harbouring active rickettsaemias of this emerging zoonotic pathogen, and supports the role of dogs as important reservoir hosts. This finding was also supported by a study reporting concurrent *R. felis *infections in a PCR-positive dog and its infected owners in Spain [12].

In this study, the flea infestation in indigenous community dogs was not evaluated in terms of flea species and burden. However, the cat flea, *C. felis*, is the most common flea infesting dogs in Australia [7,13]. These indigenous community dogs lead a semi-domesticated life. They are mostly poorly cared for and often infested by ectoparasites. Hence, it is likely that the dogs in this study were infested with fleas.

The prevalence of *R. felis *infection in indigenous community dogs (2.3%) is lower than those reported in pound dogs in SE QLD (9%) [8]. The transportation of blood using FTA cards and different DNA extraction technique may have partially affected the prevalence detected in indigenous community dogs. This is supported by studies showing amplification of DNA extracted from whole blood is more sensitive and reliable than filter paper-based technologies [14,15]. It should also be noted that neither study was particularly comprehensive with respect to sampling across different sites in each region, hence caution is required in making detailed comparisons with respect to prevalence. In this study, all dogs also appeared physically healthy, a common feature that is usually a characteristic of reservoir hosts, however further study is needed to determine the pathogenesis and pathogenic potential of *R. felis *infections in dogs.

## Conclusion

This study further supports the potential role of dogs as reservoir hosts of *R. felis *and suggests that this pathogen has a wide geographical distribution in Australia.

## Competing interests

The authors declare that they have no competing interests.

## Authors' contributions

SFH carried out the laboratory work, data analysis, intellectual interpretation and writing of the manuscript. RJT designed the study project, supervised the study, and was involved in intellectual interpretation and critical revision of the manuscript for publication. SRK supervised the study and was involved in intellectual interpretation and critical revision of the manuscript for publication. MFT, CAO and RLR revised the article critically for important intellectual content. All authors read and approved the final manuscript.
